# Catalyst-free synthesis of sub-5 nm silicon nanowire arrays with massive lattice contraction and wide bandgap

**DOI:** 10.1038/s41467-022-31174-x

**Published:** 2022-06-20

**Authors:** Sen Gao, Sanghyun Hong, Soohyung Park, Hyun Young Jung, Wentao Liang, Yonghee Lee, Chi Won Ahn, Ji Young Byun, Juyeon Seo, Myung Gwan Hahm, Hyehee Kim, Kiwoong Kim, Yeonjin Yi, Hailong Wang, Moneesh Upmanyu, Sung-Goo Lee, Yoshikazu Homma, Humberto Terrones, Yung Joon Jung

**Affiliations:** 1 Department of Mechanical and Industrial Engineering, Northeastern University, Boston, MA USA; 2 Korea Institute of Science and Technology, Seoul, Republic of Korea; 3 Department of Energy Engineering, Gyeongsang National University, Jinju, Republic of Korea; 4 Kostas Advanced Nano-Characterization Facility, Kostas Research Institute, Northeastern University, Burlington, MA USA; 5 National Nano Fab Center, Korea Advanced Institute of Science and Technology, Daejeon, Republic of Korea; 6 Department of Materials Science and Engineering, Inha University, Incheon, Republic of Korea; 7 Institute of Physics and Applied Physics, Yonsei University, Seoul, Republic of Korea; 8 CAS Key Laboratory of Mechanical Behavior and Design of Materials, Department of Modern Mechanics, University of Science and Technology of China, Hefei, China; 9 Advanced Materials Division, Korea Research Institute of Chemical Technology, Daejeon, Republic of Korea; 10 Department of Physics, Tokyo University of Science, Tokyo, Japan; 11 Department of Physics, Applied Physics and Astronomy, Rensselaer Polytechnic Institute, Troy, NY USA

**Keywords:** Nanowires, Nanowires

## Abstract

The need for miniaturized and high-performance devices has attracted enormous attention to the development of quantum silicon nanowires. However, the preparation of abundant quantities of silicon nanowires with the effective quantum-confined dimension remains challenging. Here, we prepare highly dense and vertically aligned sub-5 nm silicon nanowires with length/diameter aspect ratios greater than 10,000 by developing a catalyst-free chemical vapor etching process. We observe an unusual lattice reduction of up to 20% within ultra-narrow silicon nanowires and good oxidation stability in air compared to conventional silicon. Moreover, the material exhibits a direct optical bandgap of 4.16 eV and quasi-particle bandgap of 4.75 eV with the large exciton binding energy of 0.59 eV, indicating the significant phonon and electronic confinement. The results may provide an opportunity to investigate the chemistry and physics of highly confined silicon quantum nanostructures and may explore their potential uses in nanoelectronics, optoelectronics, and energy systems.

## Abstract (web summary)

The preparation of quantum silicon nanowires, materials with potential application in high-performance nanodevices, is challenging. Here, the authors synthesize vertically aligned sub-5 nm silicon nanowires via a vapor phase silicon etching process; the resulting material features unusual lattice reduction and significant phonon and electronic confinement effects.

## Introduction

Silicon nanowires (SiNWs) have been actively researched in the past decades for a wide variety of topics, including nanoelectronics, optoelectronics, sensing/detection, biotechnology, and energy systems^1–9^. Notably, as the electronic devices’ physical size becomes petite, the quantum confinement effect on electronic characteristics emerges significantly^10^. Theoretical and experimental studies have confirmed that the indirect bandgap of silicon could be tailored to a direct bandgap as the diameter of SiNW approaches the carrier de Broglie wavelength (1 nm for electron)^11–13^. The bandgap of these small nanowires can be increased several eVs above the bulk value (*E*_g_ = 1.12 eV), placing its photoluminescence in the visible range^14,15^.

The current SiNW synthesis methods use catalyst-nanoparticle-assisted vapor–liquid–solid (VLS) growth^16–22^ or wet chemical etching processes^23–25^, whose diameters are constrained by the size of the catalyst nanoparticle^26,27^. As a result, the typical diameters of formed SiNWs are quite large (10–100 nm)^25,28^, where the effects of 1D confinement can at best be partial. While there were valuable successes in fabricating further reduced diameter of SiNWs through the VLS growth with small catalyst nanoparticles^27,29,30^, etching oxide sheath of core-shell SiNWs^12^, or supercritical solution-phase growth with Au nanoclusters^14^, most of these synthesis methods still use catalyst nanoparticles requiring complex purifying treatments to remove them, and which in the process damage and dope SiNWs. Moreover, the as-produced SiNWs are not aligned and in low growth density^31,32^. This might be one of the key reasons why much less is experimentally known about crystalline 1D silicon at sub-5 nm quantum-confined dimensions.

Here, we report a chemical vapor etching (CVE) process that enables the formation of highly dense and vertically aligned ultra-narrow SiNWs. Crystalline sub-5 nm SiNWs up to a few tens of micrometers in length (aspect ratios greater than 10,000) can form directly in Si wafers without any catalyst nanoparticles representing the formation mechanism is different from catalyst-aided growth and etching approaches. SiNWs produced via the CVE process reported herein exhibit extraordinary lattice reduction up to 20% and excellent oxidation stability over conventional silicon. We also report rich quantum confinement properties within a strong 1D confinement of ultra-narrow silicon nanowires.

## Results and discussion

### Morphology and formation mechanism

**Fig. 1:**
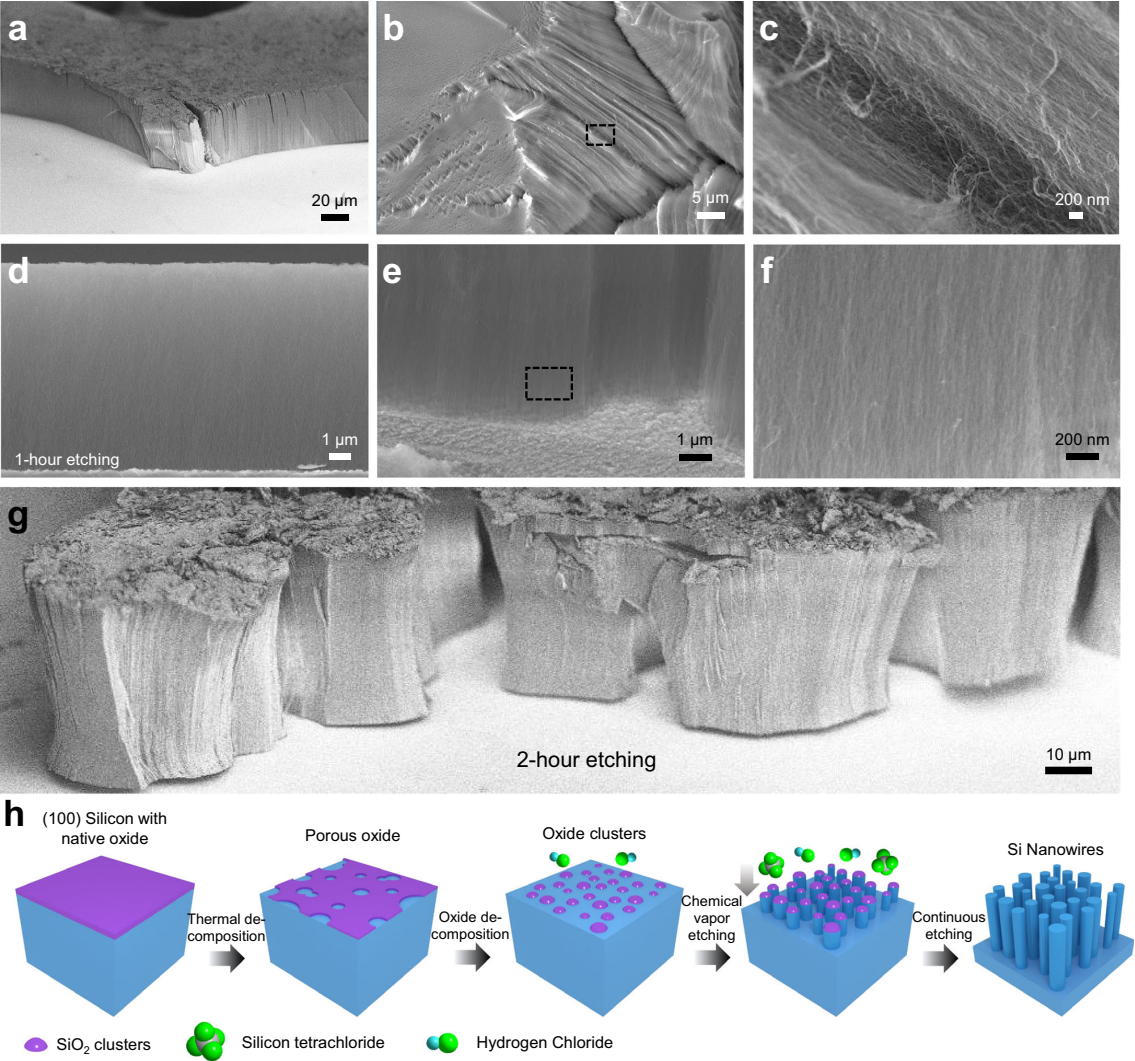
Morphology of the vertically aligned high-density SiNWs formed on the (100) silicon substrate. **a** Tilted, **b** low, and **c** high magnification planar views of the vertically aligned SiNWs. **d** SEM image of the vertically aligned SiNWs on the (100) Si wafer after 1 h etching. **e** High magnification SEM image of the bottom interface between SiNWs and etched Si substrate. **f** Enlarged image of the dashed rectangular area in (**e**). **g** Tilted views of the vertically aligned SiNWs after 2 h etching. **h** Schematic illustration of the oxide-induced etching process to fabricate ultra-narrow Si nanowires.

Scanning electron microscopy (SEM) images in Fig. 1a–c demonstrate striking examples of ultra-dense and vertically aligned SiNWs where formed arrays closely resemble high-density and vertically aligned single-walled carbon nanotube forest. The surface-directed SiNW forest is fabricated directly from the Si wafer, etching vertically on the flat surface with a 9 µm height after 1 h high-temperature CVE using SiCl_4_ gas in a highly controlled Ar and H_2_ environment (Fig. 1d and Supplementary Fig. 1). A close examination (Fig. 1e, f) at the bottom of the SiNW array shows that very narrow nanowires are densely packed with high uniformity. After 2 h etching, the highly dense and well-aligned nanowires could be further extended to a 37 µm height (Fig. 1g). These SiNWs can be dispersed in solvents such as ethanol, and isolated nanowires can be obtained through a simple sonication (Supplementary Fig. 2). Figure 1h illustrates the oxide-induced CVE process synthesizing the ultra-narrow SiNWs. According to equilibrium concentration calculations, the dominating etchants responsible for the etching of Si at 1400 K in atmospheric pressure are SiCl_4_ and HCl (Supplementary Note 1 and Supplementary Tables 1, 2). The HCl vapor etchant is first generated from SiCl_4_ decomposition with the involvement of H_2_ at around 1400 K, along with other gas compounds (SiCl_3_, SiCl_2_, and SiHCl_3_). The initial silicon substrate had a native oxide layer of 2–2.5 nm thickness (Supplementary Fig. 3a, d). As the temperature increased to 1150 °C in the Ar atmosphere, we observed the thermal desorption of the native oxide layer into 1–2 nm thickness (Supplementary Fig. 3b, e)^33,34^. When the SiCl_4_ was introduced at 1150 °C to the CVE reactor, the oxide layer was further inhomogeneously etched by HCl to a highly porous and high-density clustered oxide structure, which acted as a mask to allow a controlled number of etchants to diffuse into the Si (Supplementary Fig. 3c, f and Supplementary Table 3). From a large representative set of cross-sectional TEM images, we extract the diameter distributions (violin plots) of pores (2.55 nm on average) and oxide clusters (1.92 nm on average) (Supplementary Fig. 3g).

The Si substrate is then etched into nanowire structures by the two major anisotropic etchants, SiCl_4_ and HCl vapors, using porous Si oxide as a mask, producing byproducts SiCl_2_, SiHCl_3,_ and H_2_ at the same time. The continuation of the above CVE process leads to the elongation of well-aligned nanowires. We speculate that with the increase of oxidant concentration (>5.5 ppm), the oxidant gases will oxidize and passivate the surface of SiNWs, facilitating nanowires’ survival from the etchant vapors. The emergence of high-density nanowires will decrease the concentration of etchants penetrating the nanowire array and alleviate the lateral etching of the nanowires, resulting in the formation of an extended nanowire array (Supplementary Fig. 4a). However, when the concentration of oxidant gas is above the threshold, the whole Si surface will be oxidized, leading to a continuous dense oxide film resistant to etching (Supplementary Fig. 4b). Furthermore, we found that the dominant etching of Si without nanowire formation occurred under very low O_2_/H_2_O concentration (<5 ppm) (Supplementary Fig. 4c).

### Crystal structure analysis

**Fig. 2:**
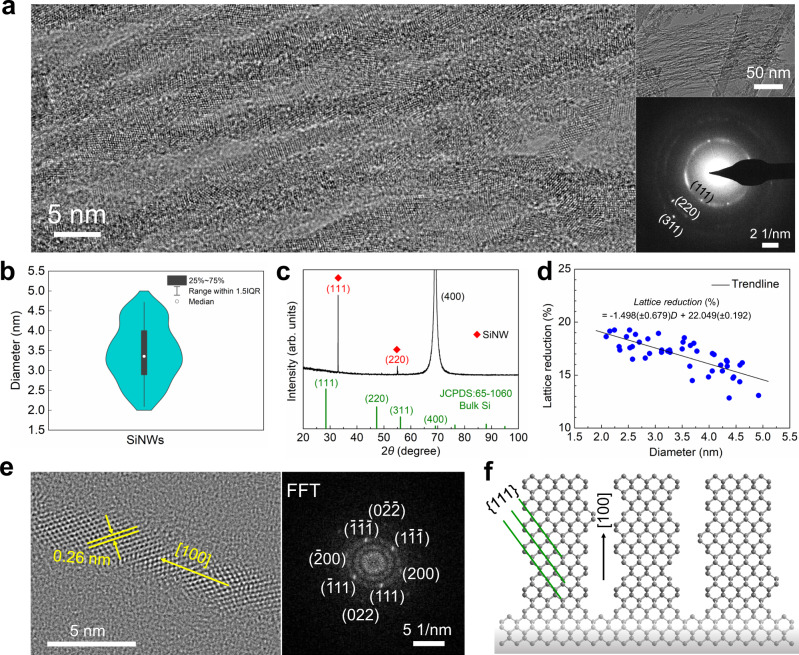
Crystal structure analysis of the vertically aligned high-density SiNWs. **a** HRTEM images and SAED pattern of multiple crystalline SiNWs. **b** Violin plot showing diameter distribution as determined from HRTEM. **c** X-ray diffraction pattern of the vertically aligned SiNWs on (100) Si substrate. The strong (400) peak is from the (100) silicon wafer substrate. **d** The relationship between lattice reduction and the diameter of the SiNWs. **e** HRTEM and corresponding FFT images of an individual Si nanowire. **f** Schematic illustration of the crystal orientation of vertically aligned (100) SiNWs on (100) Si substrate observed from <110> direction with a relatively rough edge.

High-resolution transmission electron microscopy (HRTEM) was used to obtain direct evidence of the crystalline structure of the vertically aligned SiNWs with sub-5 nm diameters and lengths up to several micrometers. An HRTEM image of the SiNW bundle in Fig. 2a shows clear lattice fringes of the SiNWs, reflecting the highly crystalline nature without significant oxidation on the nanowires’ surface (Supplementary Note 2 and Supplementary Fig. 5). The nanowires’ structural features have been further studied by the selected area electron diffraction (SAED) pattern. The diffraction rings from randomly-dispersed SiNWs can be used to index the structure, and each radius corresponds exactly to the interplanar distance *d*_*hkl*_. The observed three diffraction rings at about 2.50, 1.53, and 1.31 Å reveal the presence of {111}, {220}, and {311} planes of the diamond cubic lattice (space group *Fd3m*)^35^. The calculated lattice parameter is 4.33 Å, which is 79.7% of that for bulk Si (5.43 Å). The inordinately isotropic large compression of the diamond cubic Si lattice under atmospheric pressure and room temperature is surprising. By analyzing a large representative set of HRTEM images (Fig. 2b), we extract the diameter distribution (violin plot) of nanowires in the range of 2–5 nm (3.44 nm on average) with narrow diameter size distribution (relative standard deviation ~20.7%). The majority of the nanowires show a diameter comparable to or smaller than the exciton Bohr radius (~5 nm)^36,37^, a size range of quantum confinement effects for tunable electronic and optical properties. To corroborate the extraordinary lattice reduction, we have performed X-ray diffraction (XRD) analysis on the SiNW sample. XRD pattern of the vertically aligned SiNWs on (100) Si substrate shows the 2*θ* shift of {111} plane from 28.45° (bulk Si, JCPDS card No. 65–1060) to 32.96°, as well as that of (220) plane from 47.31° to 54.99° (Fig. 2c). The strong (400) peak at 69.12° arises from the (100) silicon wafer substrate. The corresponding lattice constant of SiNWs is around 4.70 Å, reconfirming the lattice reduction (13.4%).

Past studies have shown that etching rates in silicon wafers are sensitive to local strain^38^. Specifically, tensile strains result in significantly faster etching rates^38^. We hypothesize that sustained nanowires formation following initial etching, therefore, requires exposed surfaces with tensile strains, that are stabilized by lattice contraction of the bulk lattice. The large lattice contraction we observe can arise due to the increasing need for tensile strained surfaces as the nanowire size decreases. For low-dimensional crystals, surface stresses and related surface reconstructions (e.g., dimerization in silicon) can lead to residual strains that can become significant as the size approaches a few nanometers^39–42^. In extreme cases, they can result in non-linear elastic deformations, superelasticity, and even lattice phase transformations^43–45^. Size dependence of the lattice deformations is a signature of these effects, and to see if the contraction we observe is correlated with the nanowire size, we analyze several HRTEM images of SiNWs along their length. Figure 2d shows that the lattice reduction (%) increases with the decrease of local nanowire diameter, *D*, and the increase is approximately linear, that is

1 $${{{{{\rm{Lattice}}}}}}\,{{{{{\rm{reduction}}}}}}\;( \% )=-1.498\;(\pm 0.679)D+22.049\;(\pm 0.192)$$

with the net reduction in the range of 13–20%. Note that there is a considerable fluctuation in the measured lattice contraction at a fixed diameter, which may be attributed to the wiggled morphology of the randomly-positioned nanowires. In addition, the etchants aggressively attack and remove the surface Si atoms, which causes a slightly non-uniform diameter of the nanowire and also contributes to the complexity in evaluation. The size dependence is suggestive of a surface effect as the origin of the stabilization of the lattice contraction, suitably aided by a stress-induced etching pathway for the formation of Si surface nanowire clusters and atomically thin Si suboxide sheath that is amplified as nanowire diameter approaches a few nanometers^46,47^ (Supplementary Fig. 5). The combination of XRD characterization, TEM measurements of lattice fringes along with the axial and radial directions, and size dependence of the contraction show that the nanowire unit cell remains cubic upon contraction. The nanowire is therefore uniformly compressed along with both axial and radial (and therefore also azimuthal) directions, with the resultant strains scaled by the Poisson’s ratios. We attribute this hydrostatic stress state to the etching reaction chemistry, likely stabilized by the oxide layer that forms in the wake of the surface reactions, and delegate a more detailed analysis of the compressed nanowire stability to a later study.

To probe further, the HRTEM images recorded along the [110] zone axis of crystalline SiNWs and perpendicular to the nanowire’s long axis reveal fringes from {111} planes. HRTEM of multiple individual nanowires shows the vertically aligned SiNWs with corresponding interplanar spacing *d*_*111*_ of around 0.26 nm (Fig. 2e and Supplementary Fig. 6). The corresponding fast Fourier transform (FFT) images reveal the nanowire formation direction to be [100], which is the same orientation of the initial silicon wafer. This etching technique shows small dependence upon the doping characteristics of the Si substrates, i.e., SiNWs can be derived from n-type, p-type, and highly doped p-type Si wafers (Supplementary Fig. 7). As shown in Fig. 2e, the atomic-scale roughness of the surface of SiNW is commonly observed. The rough morphology of the SiNW sidewalls may cause the scattering of electrons and/or phonons^48^. The anisotropic etching of the Si surface depends on the removal rates of Si atoms associated with the back-bond strength theory^49,50^. Specifically, the {100} surfaces have only two bonds connected to the substrate while the other surfaces, such as {111} surfaces, have three. The weakening effect of the Si back bonds due to the bonding of the surface Si atoms to Cl^−^ is more substantial for {100} surfaces. In addition, the surface energies of Si {100}, {110} and {111} are 1.99, 1.41 and 1.15 J cm^−2^, respectively^51^. Si {100} plane with higher surface energy and surface bond density (1.36 × 10^15^ cm^−2^) provides more reacting points, causing a much faster Si atom removal rate and the anisotropic <100> etching (Fig. 2f).

### Optical properties and bandgap

**Fig. 3:**
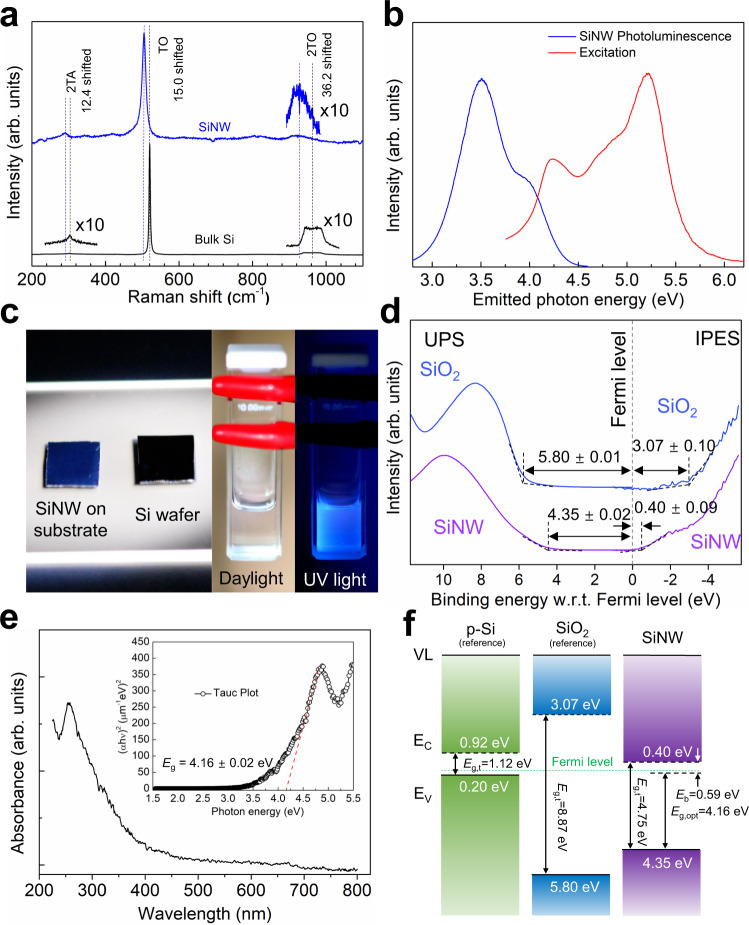
Optical properties and bandgap of the vertically aligned ultra-narrow SiNWs. **a** Raman characterization result showing clear SiNW peaks with redshift. **b** Photoluminescence spectrum of ultra-narrow dispersed in ethanol with excitation energy of 5.17 eV. **c** Photographs of the as-fabricated SiNWs on Si and SiNWs dispersion in ethanol under UV light. **d** Combined UPS and IPES spectra show the quasi-particle bandgap of SiNWs with the conduction/valence band offsets. **e** UV–vis absorption spectrum of SiNWs. Tauc plot indicates the direct bandgap transition for SiNWs. **f** Energy-level diagram of the SiNWs derived from UPS, IPES, and PL. *E*_*g,t*_, *E*_*g,*opt_ and *E*_*b*_ are transport bandgap, optical bandgap, and binding energy, respectively.

Raman spectroscopy with an excitation wavelength of 532 nm was used to distinguish the structural fingerprint for ultra-narrow SiNWs. All measurements were performed at low laser power and room temperature to eliminate the heat effects (Supplementary Figs. 8, 9 and Supplementary Tables 4, 5). Figure 3a compares the Raman spectra of bulk Si and SiNWs characterized at the same excitation energy but with different exposure times. The bulk crystalline Si shows a representative Raman peak at 520 cm^−1^ due to the scattering of the first-order optical phonons^52^. However, in our SiNWs, the corresponding Raman peak was redshifted by almost 15 cm^−1^ compared to bulk Si. Its line-width broadened (FWHM of 12.4 cm^−1^), and the line shape became asymmetric^52–55^. The redshift of the second-order spectra of transverse acoustical phonon mode (2TA, from 302 to 290 cm^−1^) and transverse optical phonon mode (2TO, from 969 to 933 cm^−1^) have also been observed in SiNWs. Such wavenumber downshifts of Raman peaks can be mainly attributed to the phonon confinement effects dominated by diameter reduction^52–54,56^. When the crystal size is reduced to the nanoscale, the momentum conservation rule is relaxed, and the phonon scattering is not limited to the center of the Brillouin zone, while phonon dispersion near the center will also be considered^56^. The smaller the crystal size is, the bigger the wavenumber downshifts, and the more asymmetric and broader the Raman peak becomes^53^.

It is known that as the diameter of SiNWs approaches the carrier de Broglie wavelength, the bandgap of SiNW is renormalized due to quantum confinement effects^11,57^. It is also expected that sub-critical diameter SiNWs exhibit a direct bandgap^58^, which increases as the nanowire diameter decreases regardless of surface termination^13^. As the diameter narrows, the nanowire’s bandgap gradually widens and deviates from that of bulk Si^12^. As shown in Fig. 3b, using a 240 nm excitation wavelength (5.17 eV) at room temperature, the ultra-narrow nanowires show strong photoluminescence (PL) peak-centered at 3.50 eV and a weak shoulder peak at 3.8 eV. With respect to the bulk crystalline Si indirect bandgap of 1.12 eV^59^, the PL peak has been significantly blue-shifted, which can evidence the renormalized bandgap of SiNWs. In Fig. 3c, it is confirmed that the blue light emissions under UV light (4.88 eV) of both as-fabricated SiNWs on the Si substrate and SiNWs dispersed in ethanol corresponds to the emission energy of the PL spectrum.

Ultraviolet photoemission spectroscopy (UPS) and inverse photoemission spectroscopy (IPES) were employed to investigate the density of state (DOS) of the ultra-narrow SiNWs and their energetic position of valence band maximum (VBM) and the conduction band minimum (CBM). To identify the feature of the DOS of ultra-narrow SiNWs and their surface oxidation, native SiO_2_ was measured as a reference. In both cases, apparent O 2*p* valence features were observed around 7–9 eV for UPS and −3 ~ −4 eV for IPES in Fig. 3d. From that, the DOS of inside SiNWs was obtained, and their level onsets centered around the Fermi level were found at the binding energy of 4.35 eV for the VBM and 0.40 eV for the CBM, indicating a quasi-particle bandgap of 4.75 eV for the ultra-narrow SiNWs. The combination of UPS and IPES spectra reconfirm bandgap renormalization as it directly indicates the DOS of SiNW. Moreover, we couldn’t observe noticeable features between VBM and CBM onset, possibly suggesting the very low density or absence of in-gap state stems from the defect or impurities within the margin of experimental error^60–63^. However, further investigation should be followed for a comprehensive understanding of defects and impurities within SiNWs. To determine the optical bandgap energy, the Tauc plot (Fig. 3e, Supplementary Note 3) has been obtained from the UV–vis absorption spectrum. A direct optical bandgap of 4.16 eV has been extracted for the SiNWs by extrapolating the linear region to the abscissa of photon energy. Besides, in Fig. 3f, the large exciton binding energy of 0.59 eV, which is one of the fingerprints of quantum confinement effects, was estimated by comparing the optical bandgap of SiNWs derived from the absorption spectrum with the quasi-particle bandgap (${E}_{b}={E}_{g,t}-{E}_{g,{{{{{\rm{opt}}}}}}}$). Surprisingly it is ca. 100 times higher than that of bulk Si (0.0055 eV)^64^. We assume that these largely increased quasi-particle bandgap of 4.75 eV, and exciton binding energy of 0.59 eV are might be due to a combination of different factors, including the highly effective quantum confinement dimension^11,57^, extraordinary lattice reduction (energy gap increase due to interatomic distance reduction)^65–68^, and dielectric screen effect from Si/SiO_*x*_ core-shell structure of SiNW^65,66^.

### Stability against oxidation

**Fig. 4:**
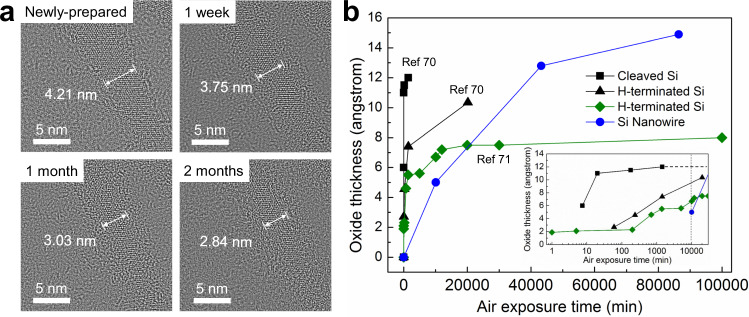
Stability against oxidation of the vertically aligned ultra-narrow SiNWs. **a** HRTEM images showing the stability of the ultra-narrow Si nanowire subject to air exposure at room temperature. Note that the identical SiNW was observed in HRTEM for different time durations. The sizes of the crystalline Si cores are measured. **b** Oxide thickness as a function of exposure time to ambient air^70,71^.

To investigate the stability of SiNWs in the air, SiNWs were exposed to ambient air (room temperature 22 °C, relative humidity 40–50%) for up to two months without any surface modification/termination, and HRTEM images of SiNWs have been recorded accordingly (Fig. 4a). The newly prepared SiNWs show clear lattice fringes without a noticeable amorphous oxide shell. After 7, 30, and 60 days in the air, the same SiNW surface was slowly oxidized, and the estimated oxide thickness (and corresponding measured silicon core diameter) were 5.0 Å (3.75 nm), 12.8 Å (3.03 nm), and 14.9 Å (2.84 nm), respectively. As shown in Fig. 4b, the surface oxidation rate of SiNWs decreases with time, possibly due to a self-limiting oxidation effect caused by the Si/SiO_*x*_ interface^46,47,69^. It has been reported that when bulk Si was cleaved in and exposed to air (25 °C, relative humidity 30–50%), the surface of silicon was oxidized immediately, forming 11–13 Å thickness of silicon oxide within 24 h^70^. Further, hydrogen passivated silicon also showed surface oxidation up to 7.6 Å after 24 h and 11 Å within two weeks in the air^70^. During the first seven days in ambient air, the average oxide growth rate of our ultra-narrow SiNWs was around 0.7 Å per day, which is at least 58%^70^ and 25–43%^70,71^ lower than the oxidation rate of bulk and hydrogen-passivated Si, respectively.

In this study, we demonstrated a catalyst-free synthesis of high-density, aligned, and sub-5 nm SiNWs by developing a vapor phase silicon etching process. The SiNWs are oriented along [100] direction, with a significant lattice reduction of 13–20%, which possibly enhanced the nanowires’ stability against etching and oxidation. These sub-5 nm SiNWs with extraordinary lattice reduction manifest significant phonon and electronic confinement effects, which might be beneficial for potential applications in nanoelectronics and optoelectronics, such as transistors^72,73^ and biosensors^3,74^. Furthermore, macroscopic films containing billions of such small SiNWs in a highly aligned fashion can also be a promising material system for gas/chemical sensors^75^, anodes in lithium-ion^5,76^ and lithium–sulfur batteries^77^, and solar cells^78–82^, where strong quantum confinement, as well as ultra-high surface area of silicon, is desirable.

## Methods

### Materials preparation

The fabrication of high density and well-aligned SiNWs with the sub-5 nm diameters and up to a few tens of micrometers in length was accomplished on Si wafers by developing a chemical vapor silicon etching process that uses silicon tetrachloride (SiCl_4_) gas in a highly controlled argon (Ar) and hydrogen (H_2_) environment at 1000–1150 °C. The Si wafer was located at the center of a furnace. In a typical fabrication process, the reaction chamber was pumped down to a base pressure at the order of 10^−2^ Torr and backfilled with ultra-high purity Ar gas (500 sccm) until atmospheric pressure. When the chamber’s temperature reaches 1000–1150 °C, SiCl_4_ vapor was introduced to the chamber by bubbling 20 sccm of 5–10% H_2_-balanced Ar gas through a glass bubbler. After etching, 500 sccm flow of ultra-high purity Ar gas was streamed through the chamber to help cool the chamber down.

### Materials characterizations

The characterization of SiNW morphology was conducted using a thermal field emission-scanning electron microscope (Supra 25 FE-SEM, Zeiss). For the structural analysis, an aberration-corrected transmission electron microscope (TEM, FEI Titan Themis 300) was used to obtain a direct crystalline silicon structure. The nanowires’ chemical composition was determined by the energy dispersive X-ray spectrometer (EDS) attached to TEM. SiNWs were dispersed in ethanol (Reagent Alcohol, anhydrous, ≤0.005% water, Sigma-Aldrich) and dropped on an ultra-thin carbon layer (3 nm) supported by lacey carbon and copper grid (Ted Pella, inc.). The TEM cross section samples were prepared by using SEM/focused ion beam (FIB) dualbeam system with Ga ion beam. To protect the top surface of the cross section sample, a 200 nm-thick layer of Pt was deposited by electron beam first and followed by a 2 μm-thick layer of ion beam Pt deposition. 30 kV Ga ion beam was used to make the rough cut and cleaning cross section patterns. 5 kV Ga ion beam was used for the final cross section polishing. High-resolution TEM (HRTEM) images were taken at 300 kV, and EDS mappings were performed in STEM mode. To investigate nanowires’ oxidation resistance in air, HRTEM images were recorded on the same nanowires after exposing the above grid to ambient air for different periods from 1 week to 2 months. ImageJ was used for background noise reduction of the HRTEM images. The XRD spectrum of SiNWs on Si substrate was recorded by a high-resolution X-ray diffractometer (Rigaku SmartLab) with Cu K_α_ radiation (λ = 1.54 Å) using theta-2theta scans. X-ray photoelectron spectroscopy (XPS) was performed on the nanowires with an XPS spectrometer (Thermo Fisher Scientific K-Alpha^+^). SiNWs were first dispersed in ethanol and then dropped on a highly oriented pyrolytic graphite (HOPG) substrate. The optical measurements were performed using a Raman spectroscope (Jobin Yvon HR800, Horiba) and photoluminescence (Hitachi F7000 fluorescence spectrophotometer). For the Raman sample, a stainless-steel razor blade was chosen as the substrate, which does not have any strong peaks near typical Si peaks at around 300, 520, and 960 cm^−1^. Then SiNWs were collected from the surface of the etched Si substrate, dispersed inside of ethanol using a weak sonication process, and placed on the substrate for Raman measurement. To reduce the effects of heating by the laser Raman scattering data were collected in the region of low laser flux *P* = 1.87 mW where the shape of the band is independent of *P*. For the PL sample, SiNWs were dispersed in ethanol by sonication and excited at a wavelength of 240 nm. The light-emitting examination was carried out on SiNW dispersion in a UV quartz cuvette under a UV light source (UV lamp, 254 nm) in a dark room. SiNWs on the Si substrate and SiNW dispersion in ethanol solvent were both used as samples, with Si wafer as a reference. UPS measurements were conducted using PHOIBOS 150 hemisphere (SPECS GmbH) with a He I (*hν* = 21.22 eV) discharging lamp as an excitation source. The IPES was performed in the isochromatic mode using a low-energy electron gun with a BaO cathode and a band-pass filter of 9.5 eV (SrF_2_ + NaCl). The resolution of UPS of 100 meV and IPES of 750 meV were determined by measuring the Fermi edge of a clean Au film. The binding energy of the presented spectra was calibrated with respect to the Fermi level. To avoid sample charging, the SiNWs were prepared on top of a HOPG substrate by spin-coating methods via ethanol dispersion. The transmittance of the nanowires was measured by the JASCO V-770 UV–vis spectrophotometer. To prepare the sample, a thin SiNWs film was fabricated by drop-casting ethanol dispersion on a quartz substrate, the signal of which was later subtracted from the spectrum.

## Supplementary information

Supplementary Information

## Data Availability

The data that support the findings of this study are available from the manuscript, its supplementary information, or from the corresponding author upon request. Source data are provided with this paper.

## Source data

Source Data
