# Supplementary material for: Catalyst-free synthesis of sub-5 nm silicon nanowire arrays with massive lattice contraction and wide bandgap
Source: Nat Commun. 2022 Jun 20;13:3467. doi: 10.1038/s41467-022-31174-x (PMC9209539; doi:10.1038/s41467-022-31174-x)
Supplement: Supplementary file 1 — Supplementary Information [file 41467_2022_31174_MOESM1_ESM.pdf]

# Supplementary information

## Catalyst-Free Synthesis of Sub-5 nm Silicon Nanowire Arrays with Massive Lattice Contraction and Wide-Bandgap

Sen Gao[1]†, Sanghyun Hong[1]†, Soohyung Park[2], Hyun Young Jung[3], Wentao Liang[4], Yonghee Lee[5], Chi Won Ahn[5], Ji Young Byun[2], Juyeon Seo[1], Myung Gwan Hahm[6], Hyehee Kim[1], Kiwoong Kim[7], Yeonjin Yi[7], Hailong Wang[8], Moneesh Upmanyu[1], Sung-Goo Lee[9], Yoshikazu Homma[10], Humberto Terrones[11] and Yung Joon Jung[1, 4]*

†These authors contributed equally to this work.

*Corresponding author. Email: y.jung@northeastern.edu (Y.J. Jung)

**Supplementary Note 1 Thermodynamical analysis of chemical vapor Si etching**

In the chemical vapor etching of Si for SiNWs fabrication, it is expected that equilibrium calculations will provide valuable information, including the major reactions, major etchants, and major products with the corresponding equilibrium partial pressures. The equilibrium calculations were performed as a function of the input ratios for the following components: $SiCl_4$, $SiCl_3$, $SiCl_2$, SiCl, SiH, $SiH_4$, $Si_2H_6$, $SiHCl_3$, $SiH_2Cl_2$, $SiH_3Cl$, Cl, $Cl_2$, H, $H_2$, HCl, Si, $Si_2$, and $Si_3$ for the $SiCl_4$-$H_2$ system. Supplementary Table 1 gives the thermodynamical data for the 16 formation reactions involved in the equilibrium at the temperature 1400 K [1,2]. The system can now be estimated with the following conditions: (1) the total pressure in the system remains 1 atm, (2) the ratio $SiCl_4/H_2$ is determined by the input ratio $SiCl_4/H_2$, which is 7:1. The initial amounts of substance (in mole) for $SiCl_4$, $H_2$, Ar, and Si (s) are set to be 7, 1, 9 and 2. To obtain the information in Si etching, the equilibrium compositions before and after encountering Si were calculated using the software FactSage 7.3 [2] and compared (shown in Supplementary Table 2). The table shows that the supplied $SiCl_4$ and $H_2$ gases react with each other to form compounds such as HCl, $SiCl_3$, $SiCl_2$, and $SiHCl_3$ before arriving at Si (s, substrate). After encountering with and etching Si, the equilibrium is re-established. The partial pressures of $SiCl_4$ and HCl have decreased (or have been consumed), while the partial pressures of $SiCl_3$, $SiCl_2$, $SiHCl_3$, and $H_2$ have increased (or have been generated) during the Si etching. Therefore, the dominating Si etching reactions at 1400 K can be expressed as follows:

$$(a+b+c)SiCl_4 + (0.5a+b+c)H_2 \rightarrow aSiCl_3 + bSiCl_2 + cSiHCl_3 + (a+2b+c)HCl \quad (1)$$

$$dSiCl_4 + (2d+3e+2f+3g)HCl + (d+e+f+g)Si\ (s) \rightarrow eSiCl_3 + fSiCl_2 + (2d+g)SiHCl_3 + (1.5e+f+g)H_2 \quad (2)$$

, where *a*-*g* are the coefficients for the dominating reactants and products. This result means that $SiCl_4$ and HCl are the major etchants in the Si etching process, while $SiCl_3$, $SiCl_2$, $SiHCl_3$, and $H_2$ are the major products. The role of $H_2$ is to generate another important etchant HCl by decomposing $SiCl_4$ in the first place.

**Supplementary Note 2 X-ray photoelectron spectroscopy (XPS) and energy dispersive X-ray spectrum (EDS) analysis**

X-ray photoelectron spectroscopy was used to study the surface chemical composition and the local bonding environments of elemental Si and Si oxides in the SiNWs. The Si 2*p* spectrum was first calibrated to oxygen O 1*s* peak (532.5 eV) [3]. It has been assumed that the spin-orbit splitting is 0.60 eV, and the branching ratio is 0.5 for the Si 2*p* spectrum [4,5]. To identify the Si and various Si suboxide states in the SiNWs, peak deconvolution of the Si 2*p* spectrum was carried out through a standard curve fitting procedure, using a Shirley background subtraction followed by Gaussian-Lorentzian functions (Supplementary Fig. 5a) [6]. The fitting curve well fits the raw data of the vertically aligned ultra-narrow SiNWs, and the decomposed Si 2*p* spectrum illustrates Si $2p_{3/2}$ at 100.40 eV. The four well-resolved suboxides peaks, including $Si_2O$, SiO, $Si_2O_3$, and the stoichiometric $SiO_2$, are at 0.96 eV, 1.78 eV, 2.68 eV, and 4.07 eV higher binding energies than the Si $2p_{3/2}$ peak, respectively. By deconvolving the curve, the total area under the Si $2p_{3/2}$ and Si $2p_{1/2}$ peaks, which represents the intensity of the unoxidized Si ($I_{Si}$), accounts for 31.02% of the whole spectrum, while that of the Si-rich structures (Si, $Si_2O$, and SiO) accounts for 63.52%. The energy dispersive X-ray spectrum revealed that the nanowires are composed of silicon and oxygen with a Si:O ratio of 1:0.59 (Supplementary Fig. 5b). It agrees well with the result from XPS that a majority of Si suboxides formed on the surface of nanowires.

**Supplementary Note 3 UV-vis absorption spectrum analysis**

The Tauc plot was obtained from the UV-vis absorption spectrum to determine the optical bandgap energy following the following equation [7]:

$$(\alpha h v)^{1/r} = B(hv - E_g) \quad (3)$$

where $\alpha$ is the absorption coefficient, $h$ is the Planck constant, $v$ is the frequency, $B$ is a constant, and $E_g$ is the optical bandgap energy. Here, $r$ is 1/2 in the case of direct allowed electronic transitions.

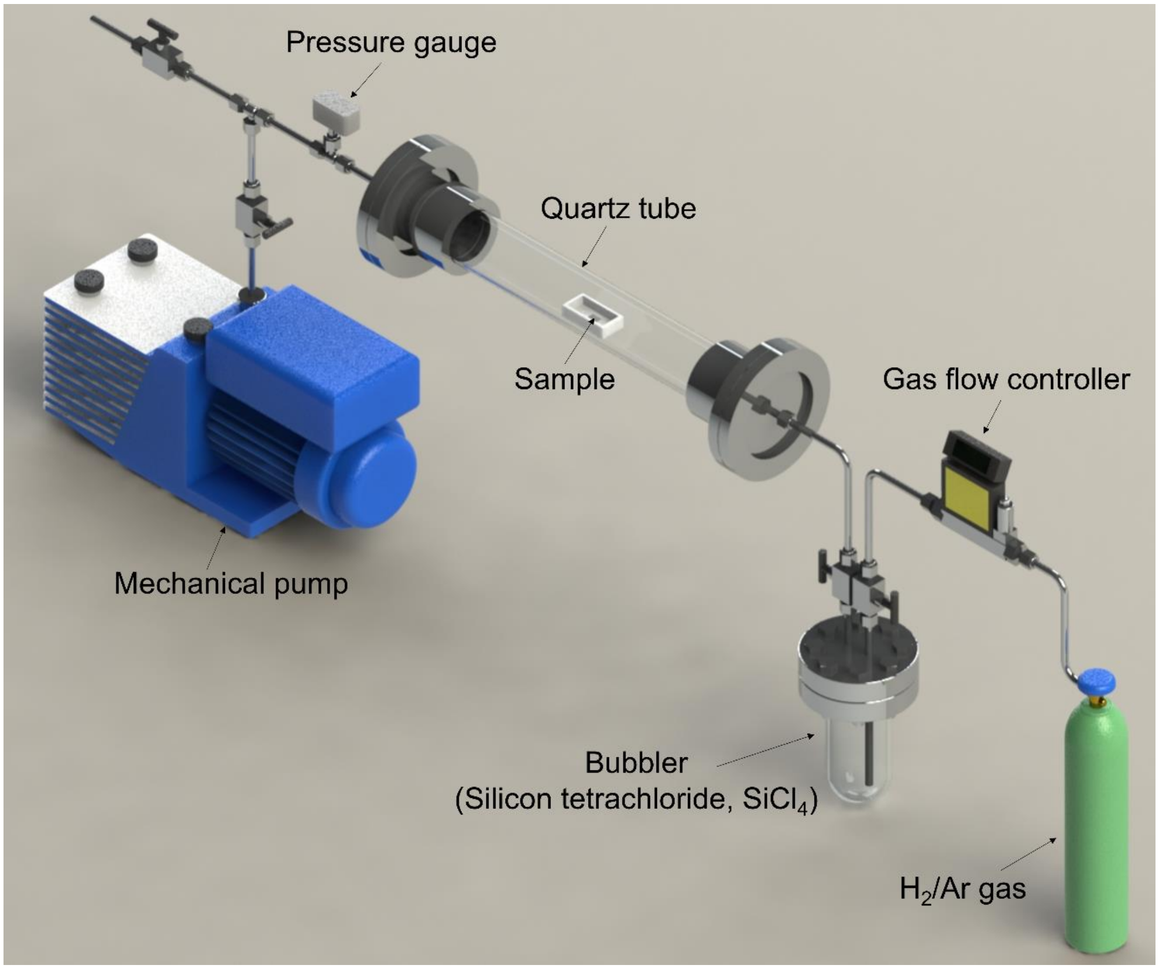


**Supplementary Fig. 1.** Experimental setup for ultra-narrow SiNWs via chemical vapor etching.

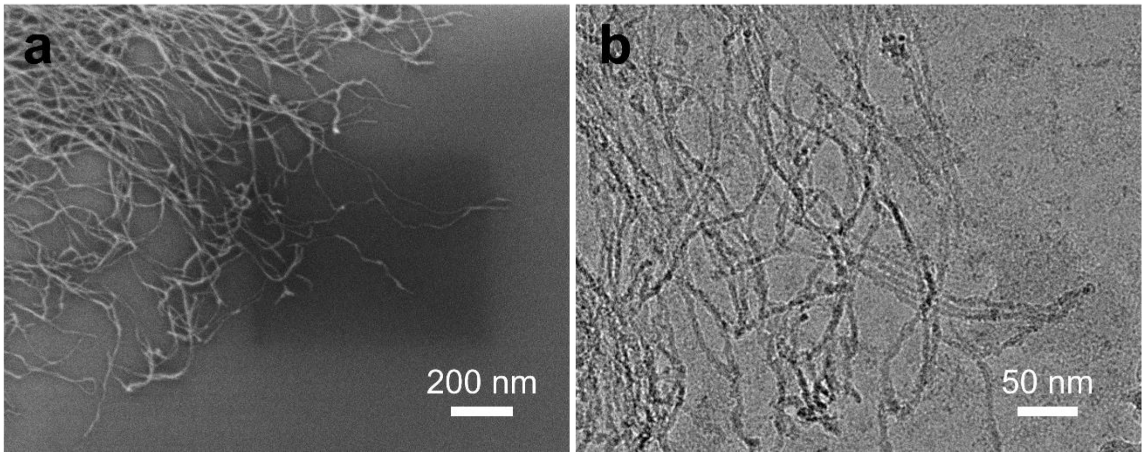


**Supplementary Fig. 2. Dispersed SiNWs on substrate. a,** SEM image of dispersed SiNWs on $SiO_2$ substrate. **b,** TEM image of dispersed SiNWs on an ultra-thin amorphous carbon film.

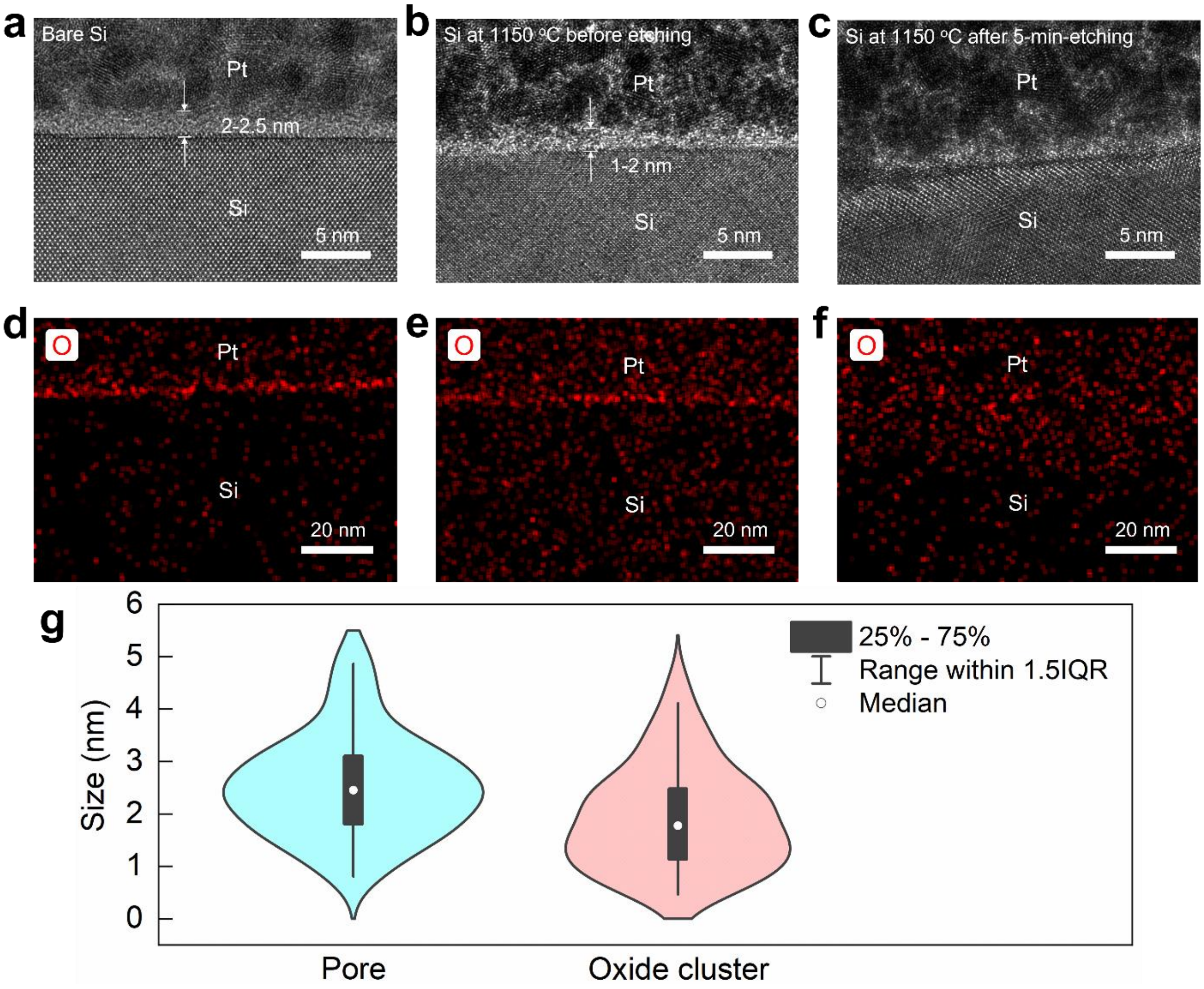


**Supplementary Fig. 3. Si substrate surface characterizations.** Cross-sectional TEM images of **a,** Bare Si, **b,** Si substrate before etching at 1150 °C, and **c,** Si substrate after 5-min-etching at 1150 °C. Oxygen elemental mapping of **d,** Bare Si, **e,** Si substrate before etching at 1150 °C, and **f,** Si substrate after 5-min-etching at 1150 °C. **g,** Violin plot showing pore and oxide cluster size distributions as determined from Cross-sectional TEM images of Si substrate after 5-min-etching at 1150 °C.

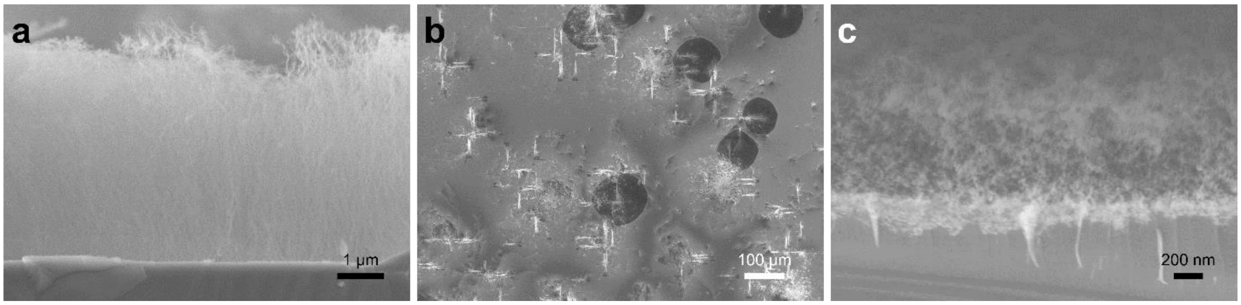


**Supplementary Fig. 4. SiNWs formation dependence on oxidant gases. a,** Cross-sectional view of Si nanowires formed under the optimal concentration of oxidant gases. **b,** Planar view of oxidized Si surface under an excessive amount of oxidant gases after etching. **c,** Cross-sectional SEM image of low-density, short Si nanowires formed under the insufficient concentration of oxidant gases.

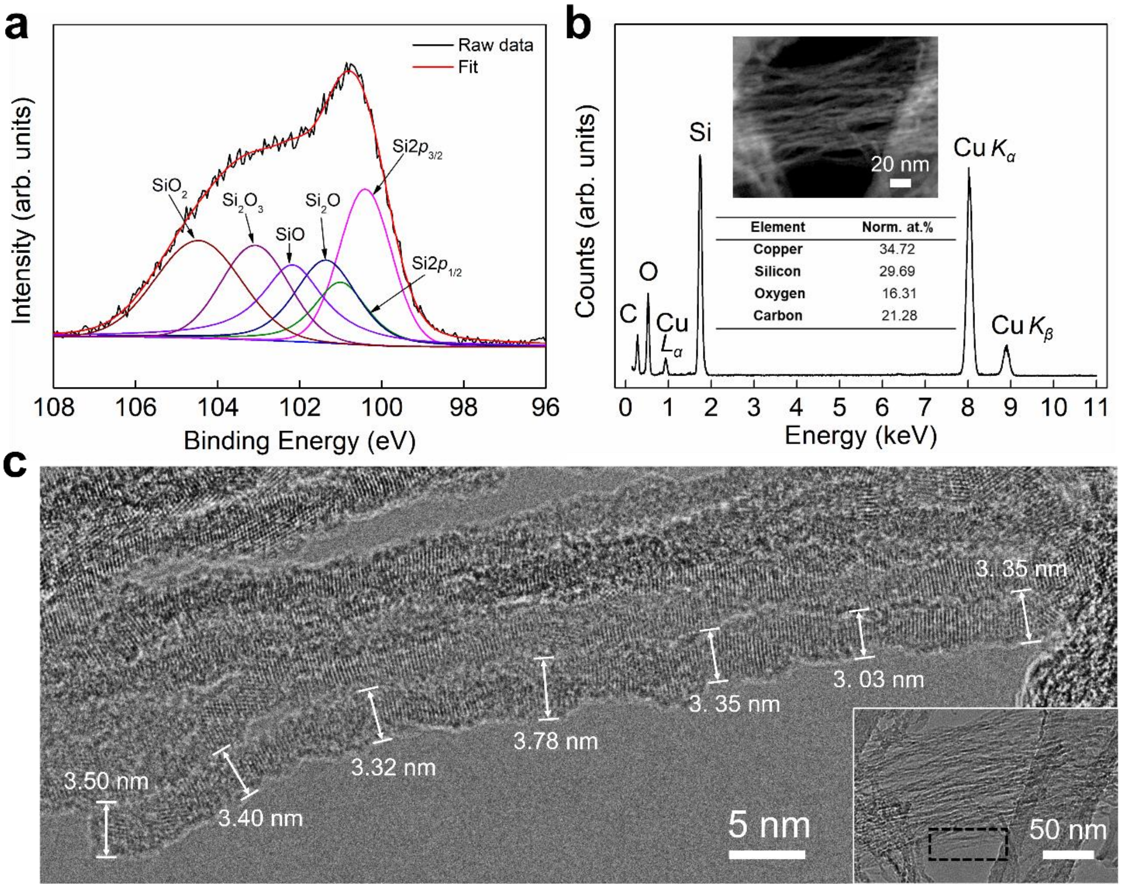


**Supplementary Fig. 5. X-ray photoelectron spectroscopy analysis. a,** XPS Si 2*p* spectrum of the vertically aligned ultra-narrow nanowires: The black curve represents the raw data, and the red line designates the entire fitted curve for the Si 2*p* core level. The four Si oxidation states, $Si^{1+}$, $Si^{2+}$, $Si^{3+}$, and $Si^{4+}$, are resolved. **b,** EDS spectrum of the Si nanowires (image shown in inset). **c,** HRTEM images of nanowires in the region where the EDS analysis is performed. X-ray photoelectron spectroscopy (XPS), the energy dispersive X-ray spectrum (EDS), and HRTEM image analysis reveals the formation of atomically thin silicon sub-oxide structures on the surface of SiNWs. The diameter of an individual nanowire was recorded for every 10 nm along the axial direction, leading to a diameter of 3.50 nm on average with high diameter uniformity (relative standard deviation ~6.4%) amid the wavy nature due to strong van der Waals interaction.

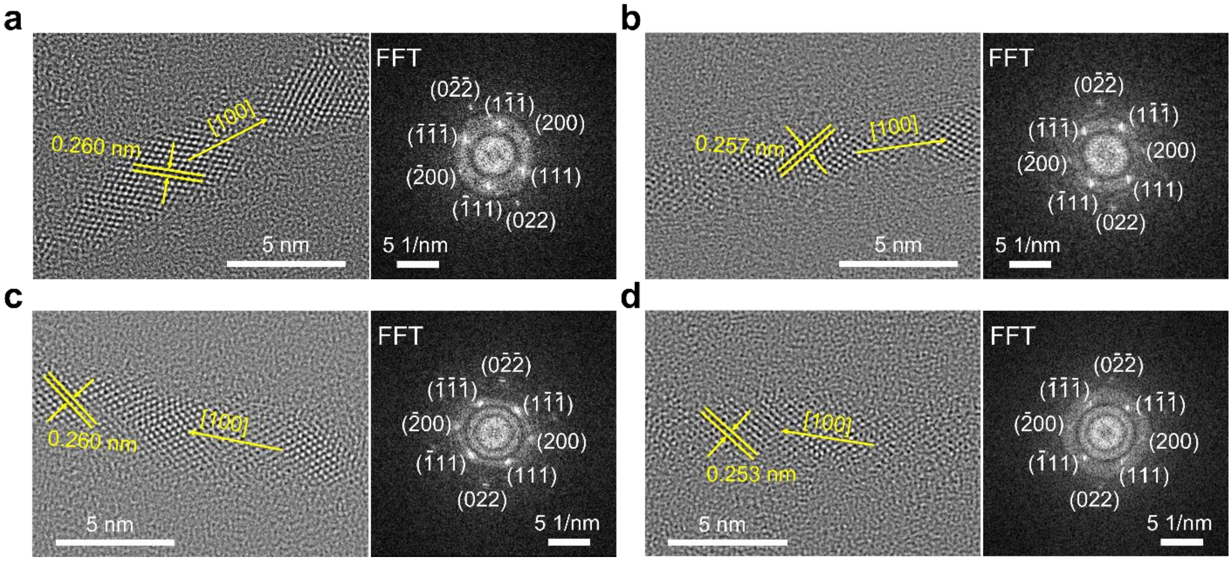


**Supplementary Fig. 6. Crystal structure of individual SiNWs. a-d,** HRTEM images and the corresponding FFT images of individual Si nanowires.

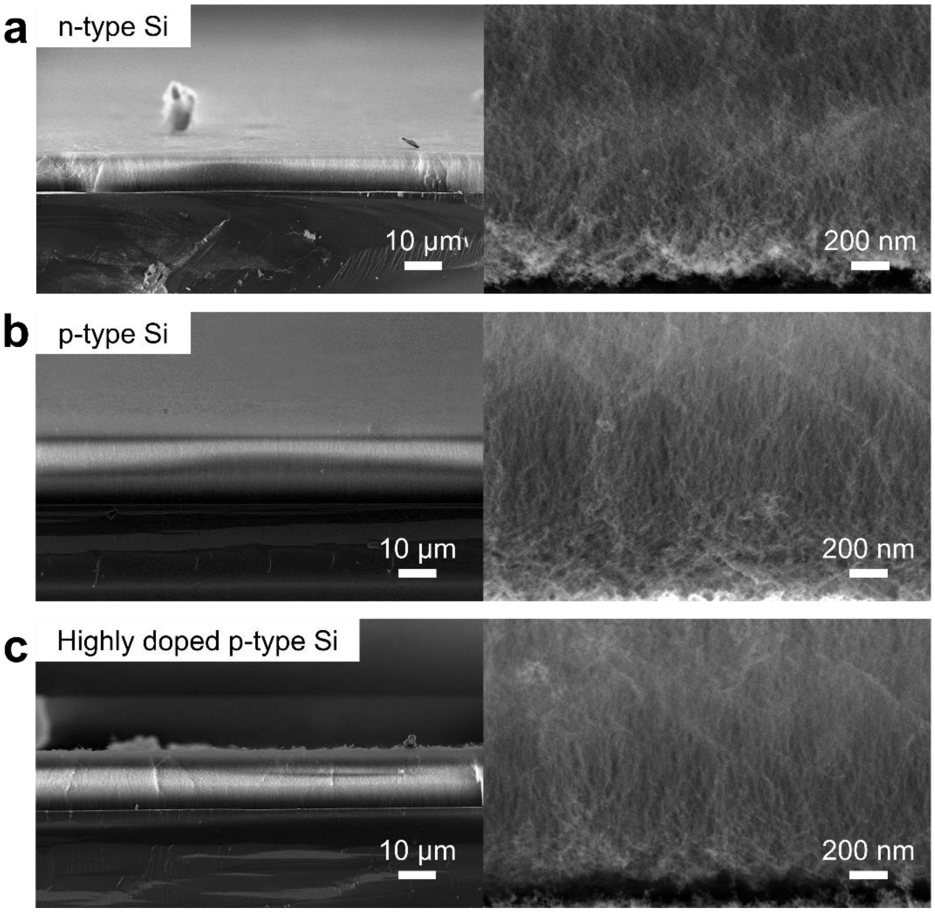


**Supplementary Fig. 7. SiNWs formation on doped Si substrates. a,** n-type, **b,** p-type, and **c,** highly doped p-type Si derived SiNWs.

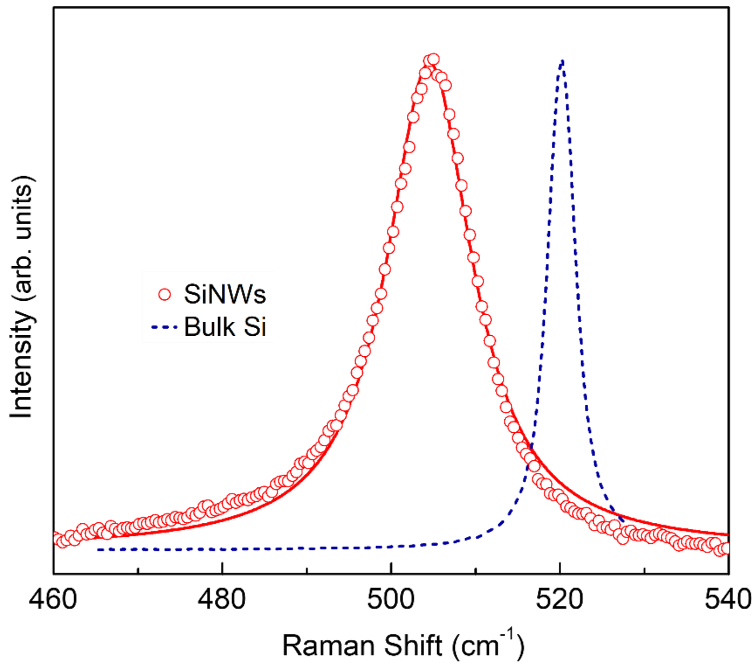


**Supplementary Fig. 8.** Comparison of first-order optical phonon spectra for SiNWs and Bulk Si. Raman scattering data were collected in the region of low laser flux *P*=1.87 mW where the shape of the band is independent of *P*, for excluding the temperature effect by the laser.

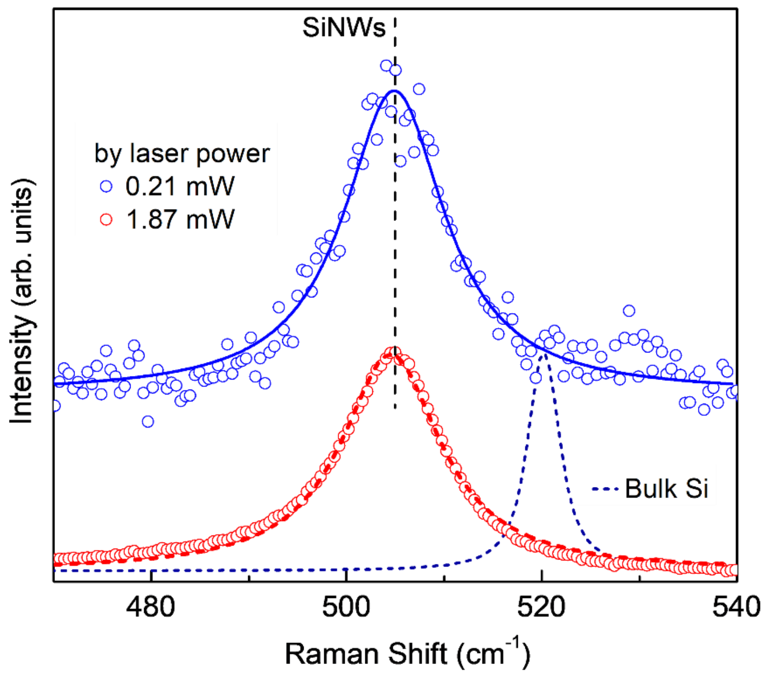


**Supplementary Fig. 9.** Comparison of first-order optical phonon spectra according to laser power.

**Supplementary Table 1.** The logarithm of the equilibrium constants of the Si-H-Cl system at 1400 K.

| Component | Formation Reaction | Equilibrium Constant | $\log_{10}K_n$ ($T$ = 1400 K) |
|---|---|---|---|
| $SiCl_4$ | Si (s) + $2Cl_2 \rightleftharpoons SiCl_4$ | $K_1 = p_{SiCl4}p^{-2}_{Cl2}$ | 17.905 |
| $SiCl_3$ | Si (s) + $3/2Cl_2 \rightleftharpoons SiCl_3$ | $K_2 = p_{SiCl3}p^{-3/2}_{Cl2}$ | 12.727 |
| $SiCl_2$ | Si (s) + $Cl_2 \rightleftharpoons SiCl_2$ | $K_3 = p_{SiCl2}p^{-1}_{Cl2}$ | 8.178 |
| SiCl | Si (s) + $1/2Cl_2 \rightleftharpoons$ SiCl | $K_4 = p_{SiCl}p^{-1/2}_{Cl2}$ | -1.970 |
| SiH | Si (s) + $1/2H_2 \rightleftharpoons$ SiH | $K_5 = p_{SiH}p^{-1/2}_{H2}$ | -8.381 |
| $SiH_4$ | Si (s) + $2H_2 \rightleftharpoons SiH_4$ | $K_6 = p_{SiH4}p^{-2}_{H2}$ | -6.030 |
| $Si_2H_6$ | 2Si (s) + $3H_2 \rightleftharpoons Si_2H_6$ | $K_7 = p_{Si2H6}p^{-3}_{H2}$ | -12.174 |
| $SiHCl_3$ | Si (s) + $1/2H_2$ + $3/2Cl_2 \rightleftharpoons SiHCl_3$ | $K_8 = p_{SiHCl3}p^{-1/2}_{H2}p^{-3/2}_{Cl2}$ | 12.905 |
| $SiH_2Cl_2$ | Si (s) + $H_2$ + $Cl_2 \rightleftharpoons SiH_2Cl_2$ | $K_9 = p_{SiH2Cl2}p^{-1}_{H2}p^{-1}_{Cl2}$ | 7.083 |
| $SiH_3Cl$ | Si (s) + $3/2H_2$ + $1/2Cl_2 \rightleftharpoons SiH_3Cl$ | $K_{10} = p_{SiH3Cl}p^{-3/2}_{H2}p^{-1/2}_{Cl2}$ | 0.720 |
| Cl | $1/2Cl_2 \rightleftharpoons$ Cl | $K_{11} = p_{Cl}p^{-1/2}_{Cl2}$ | -1.549 |
| H | $1/2H_2 \rightleftharpoons$ H | $K_{12} = p_{H}p^{-1/2}_{H2}$ | -5.315 |
| HCl | $1/2H_2$ + $1/2Cl_2 \rightleftharpoons$ HCl | $K_{13} = p_{HCl}p^{-1/2}_{H2}p^{-1/2}_{Cl2}$ | 3.851 |
| Si (g) | Si (s) $\rightleftharpoons$ Si (g) | $K_{14} = p_{Si(g)}$ | -9.082 |
| $Si_2$ (g) | 2Si (s) $\rightleftharpoons Si_2$ (g) | $K_{15} = p_{Si^2(g)}$ | -7.886 |
| $Si_3$ (g) | 3Si (s) $\rightleftharpoons Si_3$ (g) | $K_{16} = p_{Si^3(g)}$ | -6.586 |

**Supplementary Table 2.** Equilibrium partial pressures of components in the $SiCl_4$-$H_2$-Si system at 1400 K.

| | Input | Before encountering Si | | After encountering Si | | |
|---|---|---|---|---|---|---|
| **Gas Phase** | **Mole** | **Mole** | **Partial pressure** | **Mole** | **Partial pressure** | **Change** |
| **Ar** | 9 | 9.000 | 5.133E-1 | 9.000 | 4.955E-1 | - |
| **$SiCl_4$** | 7 | 6.050 | 3.450E-1 | 4.815 | 2.651E-1 | ↓ |
| **$H_2$** | 1 | 3.421E-1 | 1.951E-2 | 5.517E-1 | 3.038E-2 | ↑ |
| **HCl** | 0 | 1.192 | 6.797E-2 | 5.385E-1 | 2.965E-2 | ↓ |
| **$SiCl_3$** | 0 | 5.859E-1 | 3.341E-2 | 1.334 | 7.345E-2 | ↑ |
| **$SiCl_2$** | 0 | 2.410E-1 | 1.374E-2 | 1.570 | 8.642E-2 | ↑ |
| **$SiHCl_3$** | 0 | 1.233E-1 | 7.031E-3 | 3.503E-1 | 1.929E-2 | ↑ |
| **$SiH_2Cl_2$** | 0 | 3.781E-4 | 2.156E-5 | 3.835E-3 | 2.111E-4 | ↑ |
| **Cl** | 0 | 3.393E-5 | 1.935E-6 | 1.229E-5 | 6.765E-7 | ↓ |
| **H** | 0 | 1.184E-5 | 6.755E-7 | 1.531E-5 | 8.428E-7 | ↑ |
| **$SiH_3Cl$** | 0 | 3.335E-7 | 1.902E-8 | 1.207E-5 | 6.647E-7 | ↑ |
| **SiCl** | 0 | 2.499E-7 | 1.425E-8 | 4.657E-6 | 2.564E-7 | ↑ |
| **$Cl_2$** | 0 | 8.220E-8 | 4.688E-9 | 1.041E-8 | 5.729E-10 | ↓ |
| **$SiH_4$** | 0 | 1.207E-10 | 6.885E-12 | 1.560E-8 | 8.590E-10 | ↑ |
| **SiH** | 0 | 1.976E-10 | 1.127E-11 | 1.314E-8 | 7.236E-10 | ↑ |
| **Si (g)** | 0 | 2.813E-10 | 1.605E-11 | 1.500E-8 | 8.257E-10 | ↑ |
| **$Si_2$ (g)** | 0 | 1.292E-14 | 7.370E-16 | 3.545E-11 | 1.952E-12 | ↑ |
| **$Si_3$ (g)** | 0 | 6.148E-17 | 3.506E-18 | 8.679E-12 | 4.778E-13 | ↑ |
| **$Si_2H_6$** | 0 | 3.280E-20 | 1.870E-21 | 3.396E-16 | 1.870E-17 | ↑ |
| **Total** | 17 | 17.534 | 1.000 | 18.164 | 1.000 | - |
| **Si (s)** | 2 | 2 | - | 9.268E-1 | - | - |

**Supplementary Table 3.** Equilibrium partial pressures in the etching of silicon oxide at 1400 K.

| | Input | Before encountering Si | | After encountering Si | | |
|---|---|---|---|---|---|---|
| **Gas Phase** | **Mole** | **Mole** | **Partial pressure** | **Mole** | **Partial pressure** | **Change** |
| **HCl** | 10 | 10.000 | 1.000 | 9.978 | 0.998 | ↓ |
| **$H_2O$** | 0 | 0 | 0 | 8.674E-03 | 8.677E-04 | ↑ |
| **$SiCl_4$** | 0 | 0 | 0 | 4.334E-03 | 4.335E-04 | ↑ |
| **Cl** | 0 | 0 | 0 | 2.664E-03 | 2.665E-04 | ↑ |
| **$H_2$** | 0 | 0 | 0 | 2.218E-03 | 2.219E-04 | ↑ |
| **$Cl_2$** | 0 | 0 | 0 | 8.885E-04 | 8.887E-05 | ↑ |
| **$SiCl_3$** | 0 | 0 | 0 | 3.049E-06 | 3.049E-07 | ↑ |
| **H** | 0 | 0 | 0 | 7.202E-07 | 7.204E-08 | ↑ |
| **$SiHCl_3$** | 0 | 0 | 0 | 6.842E-08 | 6.844E-09 | ↑ |
| **OH** | 0 | 0 | 0 | 5.756E-08 | 5.758E-09 | ↑ |
| **$SiCl_2$** | 0 | 0 | 0 | 9.107E-09 | 9.110E-10 | ↑ |
| **HOCl** | 0 | 0 | 0 | 5.013E-09 | 5.015E-10 | ↑ |
| **SiO** | 0 | 0 | 0 | 1.628E-10 | 1.629E-11 | ↑ |
| **ClO** | 0 | 0 | 0 | 1.321E-10 | 1.322E-11 | ↑ |
| **$O_2$** | 0 | 0 | 0 | 3.063E-11 | 3.063E-12 | ↑ |
| **O** | 0 | 0 | 0 | 1.646E-11 | 1.646E-12 | ↑ |
| **$SiO_2$** | 0 | 0 | 0 | 6.809E-13 | 6.811E-14 | ↑ |
| **$SiH_2Cl_2$** | 0 | 0 | 0 | 1.625E-13 | 1.626E-14 | ↑ |
| **HOOH** | 0 | 0 | 0 | 1.700E-15 | 1.700E-16 | ↑ |
| **HOO** | 0 | 0 | 0 | 1.560E-15 | 1.560E-16 | ↑ |
| **$Cl_2O$** | 0 | 0 | 0 | 9.089E-16 | 9.091E-17 | ↑ |
| **SiCl** | 0 | 0 | 0 | 6.860E-17 | 6.862E-18 | ↑ |
| **$SiH_3Cl$** | 0 | 0 | 0 | 1.110E-19 | 1.111E-20 | ↑ |
| **$ClO_2$** | 0 | 0 | 0 | 2.766E-20 | 2.767E-21 | ↑ |
| **Si** | 0 | 0 | 0 | 5.609E-22 | 5.610E-23 | ↑ |
| **SiH** | 0 | 0 | 0 | 4.201E-23 | 4.202E-24 | ↑ |
| **$O_3$** | 0 | 0 | 0 | 6.593E-26 | 6.595E-27 | ↑ |
| **$SiH_4$** | 0 | 0 | 0 | 3.114E-26 | 3.115E-27 | ↑ |
| **$Cl_2O_2$(g)** | 0 | 0 | 0 | 1.247E-26 | 1.248E-27 | ↑ |
| **$Cl_2O_2$(g3)** | 0 | 0 | 0 | 2.972E-27 | 2.973E-28 | ↑ |
| **$Cl_2O_2$(g2)** | 0 | 0 | 0 | 8.802E-28 | 8.805E-29 | ↑ |
| **$ClO_3$** | 0 | 0 | 0 | 5.943E-34 | 5.945E-35 | ↑ |
| **$Si_2$** | 0 | 0 | 0 | 9.008E-38 | 9.011E-39 | ↑ |
| **$Si_2H_6$** | 0 | 0 | 0 | 3.365E-49 | 3.366E-50 | ↑ |
| **$Si_3$** | 0 | 0 | 0 | 1.499E-51 | 1.499E-52 | ↑ |
| **Total** | 10 | 10.000 | 1.000 | 9.997 | 1.000 | - |
| **$SiO_2$ (s)** | 0.1 | 0.100 | - | 9.566E-02 | - | - |

**Supplementary Table 4.** Spectral parameter of the first-order optical phonon spectra.

| **Sample** | **Diameter (nm)** | **Wavenumber shift ($cm^{-1}$)** | **FWHM ($cm^{-1}$)** | **Coefficient of broadening ($C_b$)** | **Asymmetric coefficient ($C_a$)** |
|---|---|---|---|---|---|
| SiNW | 3.44 | 15 | 12.4 | 3.26 | 1.14 |
| c-Si | - | 0 | 3.8 | 1 | 1 |

**Supplementary Table 5.** Spectral parameter of the first-order optical phonon spectra according to laser power.

| Sample | | Diameter (nm) | Wavenumber shift ($cm^{-1}$) | FWHM ($cm^{-1}$) | Coefficient of broadening ($C_b$) | Asymmetric coefficient ($C_a$) |
|---|---|---|---|---|---|---|
| SiNW | 1.87 mW | 3.44 | 15 | 12.4 | 3.26 | 1.14 |
| | 0.21 mW | | 15 | 12.3 | 3.24 | 1.04 |
| c-Si | | - | 0 | 3.8 | 1 | 1 |

**Supplementary References**


1 van der Putte, P., Giling, L. J. & Bloem, J. Surface morphology of hcl etched silicon wafers: I. Gas phase composition in the silicon hcl system and surface reactions during etching. *J. Cryst. Growth* **41**, 133-145 (1977).

2 Bale, C. W. *et al.* Reprint of: Factsage thermochemical software and databases, 2010–2016. *CALPHAD* **55**, 1-19 (2016).

3 Ma, J. W. *et al.* Carrier mobility enhancement of tensile strained si and sige nanowires via surface defect engineering. *Nano Lett.* **15**, 7204-7210 (2015).

4 Peng, C. *et al.* Stability of hydrogen-terminated surfaces of silicon nanowires in aqueous solutions. *J. Phys. Chem. C* **115**, 3866-3871 (2011).

5 Bashouti, M. Y., Sardashti, K., Ristein, J. & Christiansen, S. H. Early stages of oxide growth in h-terminated silicon nanowires: Determination of kinetic behavior and activation energy. *Phys. Chem. Chem. Phys.* **14**, 11877-11881 (2012).

6 Himpsel, F. J., McFeely, F. R., Taleb-Ibrahimi, A., Yarmoff, J. A. & Hollinger, G. Microscopic structure of the sio2/si interface. *Phys. Rev. B* **38**, 6084-6096 (1988).

7 Tauc, J., Grigorovici, R. & Vancu, A. Optical properties and electronic structure of amorphous germanium. *Phys. Stat. Sol. B* **15**, 627-637 (1966).
